# Validation of the Montreal cognitive assessment against the RBANS in a healthy South African cohort

**DOI:** 10.4102/sajpsychiatry.v24i0.1304

**Published:** 2018-09-20

**Authors:** Natalie Beath, Laila Asmal, Leigh van den Heuvel, Soraya Seedat

**Affiliations:** 1 Department of Psychiatry, Stellenbosch University, South Africa

## Abstract

**Background:**

Mild cognitive impairment (MCI) represents an intermediate state between normal cognition and dementia. Early detection and treatment of reversible contributing factors to progressive cognitive decline currently forms the cornerstone of management. As the population at risk of developing dementia is projected to increase significantly in many low- and middle-income countries where health care services continue to operate under clinical and human resource constraints, there is a need for low-cost, quick and reliable screening tools. The Montreal cognitive assessment (MoCA) was developed as a brief screening tool with high sensitivity and specificity for detecting MCI. The initial validation sample for the MoCA consisted of English and French speaking Canadians. Studies undertaken in a variety of countries show that the reliability and validity of the MoCA in screening for MCI is good; however, it has been recommended that some item modification and adjustment of cut-offs for the diagnosis of MCI in these populations may be needed to account for cultural differences.

To date, no studies have evaluated the MoCA in the South African population. We aimed to compare the validity of the MoCA to the RBANS, evaluate the effectiveness of the MoCA as a screening tool for MCI and generate normative data for the MoCA.

**Methods:**

A cross-sectional observational study comprising a sample of 370 cognitively healthy males and females aged 18 years and older of mixed race (Coloured ethnicity) who were administered the MoCA and RBANS during screening.

**Results:**

The MoCA showed acceptable internal consistency (Cronbach’s alpha of 0.624). MoCA scores were significantly associated with gender (*r* = -0.199, *p* = 0.000), and correlated with age (*r* = -0.203, *p* = 0.000) and education (*r* = 0.326, *p* = 0.000). There was a strong correlation between total scores on the MoCA and RBANS (*r* = 513; *p* = 0.000), indicating good criterion-related validity. The MoCA also showed good agreement with the RBANS according to the Bland–Altman plot. ROC statistics demonstrated that the performance of the MoCA for predicting MCI compared to the RBANS was fair with an AUC of 0.794. Using the recommended cut-off score of 26, the MoCA showed high sensitivity (94.23%) but low specificity (28.16%). When the cut-off score was lowered to 23, the sensitivity was 75% and specificity 66.77%, while a cut-off of 24 demonstrated a sensitivity of 84.62% and a specificity of 52.53%.

**Conclusion:**

Although the MoCA appears fairly reliable at identifying MCI in this population, our findings suggest that some modification to certain domains and items is needed to improve the differentiation between normal ageing and MCI. Until such time that a culturally adapted version of the MoCA has been developed and validated for this population, we suggest lowering the cut-off score to 24 in order to reduce false-positive diagnoses of MCI.

